# Cannabis, motivation, and life satisfaction in an internet sample

**DOI:** 10.1186/1747-597X-1-2

**Published:** 2006-01-12

**Authors:** Sara Smucker Barnwell, Mitch Earleywine, Rand Wilcox

**Affiliations:** 1 University of Southern California, Department of Psychology, SGM 501, 3620McClintock Avenue, Los Angeles, CA 90089-1061, USA; 2 The University at Albany, SUNY, Department of Psychology, Social Sciences 369, 1400 Washington Avenue, Albany, NY 12222, USA; 3 University of Southern California, Department of Psychology, SGM 501, 3620McClintock Avenue, Los Angeles, CA 90089-1061, USA

## Abstract

Although little evidence supports cannabis-induced amotivational syndrome, sources continue to assert that the drug saps motivation [1], which may guide current prohibitions. Few studies report low motivation in chronic users; another reveals that they have higher subjective wellbeing. To assess differences in motivation and subjective wellbeing, we used a large sample (N = 487) and strict definitions of cannabis use (7 days/week) and abstinence (never). Standard statistical techniques showed no differences. Robust statistical methods controlling for heteroscedasticity, non-normality and extreme values found no differences in motivation but a small difference in subjective wellbeing. Medical users of cannabis reporting health problems tended to account for a significant portion of subjective wellbeing differences, suggesting that illness decreased wellbeing. All p-values were above p = .05. Thus, daily use of cannabis does not impair motivation. Its impact on subjective wellbeing is small and may actually reflect lower wellbeing due to medical symptoms rather than actual consumption of the plant.

## 

The link between cannabis use and low motivation is a source of extensive debate. Anecdotal information describes the cannabis user as listless and incapable. Subsets of cannabis users demonstrating low motivation receive considerable attention in the media and among proponents of marijuana prohibition. Decades ago, researchers adopted the phrase "amotivational" to describe lethargic cannabis users. Amotivational syndrome ranks among key problems associated with the drug, and strengthens policy arguments regarding the public harm that the drug introduces [1]. The US Department of Health and Human Services [1] warns parents that youth cannabis use may result in amotivational symptoms such as an apathetic approach to life, fatigue, and poor academic and work performance. Other studies suggest that cannabis induces general apathy and an inability to progress through life successfully [2]. Yet empirical research on the effects of cannabis on users' motivation suggests a low incidence of these negative outcomes and numerous alternative explanations for their appearance [3-5].

A review of the laboratory performance research, education data and employment statistics demonstrate little support for symptoms associated with amotivational syndrome. Research on the most straightforward constructs associated with motivation (e.g. goals, focus and general productivity) fail to offer consistent evidence linking cannabis to any deficits. Some studies connect low focus and lack of goals among users with repeated cannabis use, citing possible neurological causes [2,6]. Others show that repeated cannabis use bears no effect on motivation, productivity and clarity in work tasks [7].

Investigations of other indices, including employment and education, also offer little support for amotivation. Cannabis use appears orthogonal to wages or job turnover. An examination of over 8000 people suggests that some frequent cannabis users earn higher wages than abstainers [8]. Compared to non-smokers, marijuana smokers are no more likely to be fired from their jobs [9,10]. Educational outcomes vary among frequent cannabis users. High school students using cannabis are likely to have lower grades than non-users [11], but often report using other substances that may affect grades [e.g. alcohol, other illicit drugs; [12]]. Most heavy users earned lower grades prior to their marijuana consumption, suggesting cannabis could not have caused the poorer performance [13]. College students who smoke cannabis demonstrate comparable [14] or even higher [15,16] grades than their cannabis abstinent classmates, and are more likely to pursue a graduate degree [14].

While most cannabis users do not suffer observable problems with motivation, a subset of heavy, chronic users believe that the plant impairs their drive. In several studies heavy cannabis users report that marijuana affected their motivation [17,18]. However, it is notable that other variables (e.g. comorbid drug use, baselines for low motivation) may not be examined. Long-term chronic cannabis users may demonstrate hazardous drinking behaviors [19,18]. Reilly, Didcott, Swift and Hall [18] found that perceived cannabis effects outweighed perceived effects of alcohol, a drug commonly associated with numerous negative consequences [e.g. [20]]. Moreover, many people demonstrate low motivation regardless of drug use. Duncan [21] found that 5% of over 200 students demonstrated amotivational symptoms regardless of substance use. It is possible that marijuana smokers misattribute low motivation symptoms to the plant [3].

Furthermore, not all chronic cannabis users reporting low motivation encounter the experience as negative. Compared to occasional smokers, heavy smokers in one study reported lower motivation but also higher levels of life satisfaction [17] – a primary component of subjective wellbeing [22] This result might suggest that users have different goals, but cannabis users do not have a passive and non-materialistic view of achievement compared with nonusers [23]. Thus cannabis users do not necessarily eschew traditional hallmarks of success. Yet if some heavy cannabis users derive life satisfaction outside of these motivated, culturally normative routes, then how may we understand their behaviors? Earleywine [3] ponders whether chronic users reject conventional associations among motivation, productivity and life satisfaction, and ascribe to a subculture of different values.

The extant literature addressing cannabis use, motivation and life satisfaction possess myriad problems inherent to research of this nature. Given the illegality of cannabis use, variable definitions of heavy use and abstinence, as well as problems studying individuals who lack motivation, research on cannabis and motivation proves challenging. Potential participants may hesitate to admit illegal activity. As a result, many studies on amotivational syndrome among cannabis users employ small samples [e.g. N = 5; [24]]. With smaller sample sizes, further subdivision into groups of chronic users and non-users diminishes statistical power and potential findings.

Further, researchers may define frequent cannabis use in a number of ways. Some studies may define heavy cannabis users as those who use the drug daily [e.g. [17]], while others define heavy use as twice or more per week [e.g. [23]]. Similarly, studies may define cannabis abstinence differently. One study defines abstainers as those who have not used the drug more than ten times [17]; another defines abstainers as those who have never used the drug [e.g. [25]], and numerous others rely on survey items that simply ask whether the subject currently uses cannabis. These methodological issues surrounding cannabis research (e.g. small sample size, inconsistent definition of heavy use vs. abstinence) pose possible barriers to findings. Few studies employ robust statistical methods to capture potentially small differences. Further, laboratory studies may lose potential subjects whose low motivation prevents them from traveling to the laboratory. Because of these possible confounds, supporters of amotivational syndrome argue that research finding no link between cannabis and motivation overlooks potential effects.

Despite the dearth of research supporting a link between cannabis use and low motivation, the notion persists in popular culture and academia. In the public eye, the minority of heavy cannabis users who show low motivation represents the majority. This misrepresentation introduces possible impediments to the effective treatment of cannabis abuse and dependence. We address the popular myth of the association between heavy cannabis use and low motivation in a large internet sample. The study examines self-reports of motivation among daily cannabis users and lifetime cannabis abstainers. We further seek to gauge the life satisfaction or subjective wellbeing among daily users compared to abstainers.

This study overcomes numerous methodological problems by employing a large internet sample, strict definitions of use and abstinence and robust statistical analyses. The large sample with sizeable subgroups provides adequate statistical power to detect even small effects. The strict definitions of chronic cannabis use (as daily use) and abstinence (as never trying the drug) should maximize group differences. Gathering data on the internet also serves as a potential strength. Participation does not require enough motivation to travel to a laboratory. Finally, in addition to standard statistical analyses, advanced, robust measures of inter-group differences further improve statistical power.

## Method

### Procedure

Participants responded to an email request to complete an Internet survey on cannabis use and attitudes. An initial email was sent to 200 undergraduates who had taken a course on drugs and human behavior and 100 acquaintances of the second author. The email requested that they complete the questionnaire and forward the request to others. Respondents forward the email, and approximately 1300 people replied from a variety of backgrounds, as described below.

### Measures of cannabis consumption

Participants responded to several questions regarding their cannabis consumption. An Internet consent protocol ensured confidentiality of all responses. Respondents first answered whether or not they had ever tried cannabis. Those who indicated "No" comprise the non-user group (N = 244). The remaining respondents answered questions regarding the frequency of their cannabis consumption. Those who indicated using cannabis seven days a week comprise the frequent cannabis user group (N = 243). The amount of cannabis consumed at one time varies considerably. However, limiting the user group to those who use the drug seven days per week should ensure that these individuals are regular users. Among frequent cannabis users, the median number of lifetime uses of the drug was 3000 times. The study, however, focuses on users' reports of current use to avoid hindsight bias in reporting and parallel questions on current motivation and subjective wellbeing. Almost one third (31%) of users considered themselves medical cannabis users, citing chronic pain, nausea and other medical concerns as reasons for using the drug. Whereas these individuals constitute a small but not insignificant portion of chronic cannabis users (e.g. 15% of the present sample) and their reports of health problems may only increase the likelihood of encountering differences between users and abstainers, the present study includes these individuals in analyses.

### Motivation

Respondents completed items from the Apathy Evaluation Scale [AES; [26]]. Participants responded to 12 statements regarding their own feelings of motivation on a four-point scale (e.g. Not At All; Slightly; Somewhat; Very Much). Eight items that accounted for the most variance in the scale according to scale reliability analyses were selected. Additional items were added based on face validity. See Table 2. The twelve items possessed sound internal consistency (Cronbach's α = .82). Similar measures of apathy have been used successfully in previous studies of substance use and motivation, showing increased apathy among the cocaine dependent [27]. See Table 2 for a complete list of items. Preliminary data analyses for this study indicated that higher internal consistency (Cronbach's α = .88) could be achieved when one item (I don't follow through on my plans) was removed. Internal consistency would not increase with the deletion of any other items. Thus the most reliable items measuring motivation sum to provide a composite 44-point motivation scale.

**Table 1:** Demographic Differences Among Daily Cannabis Users and Lifetime Abstainers

	Daily Cannabis Users	Cannabis Non-Users
Sample Size (N)	243	244
Gender	81% male; 19% female	51% male; 49% female
Average Age*	*M *= 37.8, *SD *= 13.3	M = 28.2, *SD *= 11.0
Maximum Income Bracket**	M = $44,431, *SD *= $23,027	M = $39,640, *SD *= $24,159

*Significant age differences emerged, *t*(473) = -8.56, *p *< .001.

**Significant income differences emerged, *t*(406) = -2.04, p < .05

**Table 2:** Apathy Evaluation Scale Items

I get things done during the day	Getting things done during the day is important to me
I approach life with intensity	Seeing a job through to the end is important to me
Getting things done on my own is important to me	I have initiative*
I have motivation*	I set goals for myself
I don't follow through on my plans (reverse coded)*	I have some interesting projects I'm working on
I'm pretty productive most of the time*	I am interested in things

*Items selected for face validity. Item total correlations range from .30 to .73.

### Subjective well-being

Respondents completed the Satisfaction with Life Scale [22]. The measure has been validated extensively, showing good reliability and convergent validity [28]. The measure includes five statements about the subject's quality of life (See Table 3). Respondents rated each statement on a seven-point scale (e.g. Strongly Disagree; Disagree; Slightly Disagree; Neither Agree nor Disagree; Slightly Agree; Agree; Strongly Agree). The removal of one item ("If I could live my life over, I would change almost nothing") increased internal consistency (α = .87) derived via item reliability analyses. Internal consistency would not increase with the deletion of any other items. Thus final measures of subjective well-being were derived from four of the five items.

**Table 3:** Subjective Well-being Scale Items

The conditions of my life are excellent	In most ways, my life is close to ideal
I am satisfied with life	So far I have gotten the important things that I want in life
If I could live my life over, I would change almost nothing	

Item total correlations range from .57 to .79.

### Robust statistical methods

Throughout the analyses, we sought to apply the best statistical techniques available. Whereas many of our analyses required means comparisons, we examined problems associated with typical tests of mean difference (e.g. T-tests). Under unequal variances or heteroscedasticity, Student's T-distribution may mask true differences even as the sample size increases [29,30]. Whereas outliers may inflate sample variances, a straightforward comparison of sample variances often does not sufficiently identify heteroscedasticity. Given the potential for heteroscedasticity and a large sample size, it is prudent to apply a method that controls for unequal variances. Welch [31] devised a comparison of means that provides accurate probability coverage and improves statistical power under conditions of heteroscedasticity.

While heteroscedastic means analysis control for unequal variances, it does not consistently control outliers. Sample means may be susceptible to inflation due to outliers [30]. Plots of motivation scores revealed outliers (e.g. data points above/below three standard deviations) even after standard data transformations (e.g. square roots). Even standard transformations of the data (e.g. square roots) may not control inflation of the mean due to outlying values. An alternative measure of location is the sample median. By effectively removing 50% of the data from each tail of the distribution, the sample median demonstrates greater resistance to inflation due to outliers and heteroscedasticity [30]. Thus the median may provide a more accurate estimation of central tendency, and a comparison of medians may offer increased statistical power. The present study employs median comparisons as an alternative way to examine central tendency.

However, other methods offer sound alternatives to overcoming outliers within the data. Wilcox [30] discusses the merits of employing a comparison of trimmed means among samples with numerous outliers. The trimmed mean refers to a sample wherein a percentage of the largest and smallest observations are removed, and the remaining values are averaged. Just as the median may offer a superior perspective by effectively removing 50% of the data from each end of the distribution, lower degrees of trimming may reveal more accurate estimates of central tendency. Trimming 20% offers numerous advantages over no trimming or the use of medians, including a smaller standard error [30]. In this case, trimming 20% of extreme values removes all outliers, leaving a relatively normally distributed sample in tact. Yuen [32] derived a method of comparing trimmed means, while employing Welch's principles for heteroscedastic means comparison. This approach can improve statistical power markedly. Thus the present study also employs comparisons of trimmed means.

Finally, the present study employs alternative measures of effect size. Effect size represents the estimated measure of the degree of separation between two distributions. Effect size estimates may further illustrate the magnitude of the difference between groups. Cohen's delta (d) effectively measures the separation of two distributions means, comparing them to a measure of standard deviation pooled between both groups. Cohen's d calculations are highly susceptible to problems associated with heteroscedasticity and non-normality – the same problems associated with standard means comparison [30]. McGraw and Wong [32] describe an alternate measure of effect size based on the probability of correctly deciding the group of origin of any randomly sampled value. The resulting value, *Q*, indicates the separation of the distributions and strength of the effect.

## Results

### Participants

Participant ages ranged from 18 to 81 (mean = 33.1, *SD *= 13.1). The group was primarily Caucasian (79%), with Asian (8%), Latino (6%), mixed-race (2%), and African-descent (2%) far behind. Over three quarters (77%) were U.S. residents from a wide array of states, while the rest were from Canada (17%), South Africa (1%), the United Kingdom (1%) and other countries throughout Europe and the Middle East (4%). Respondents varied in education (18% reported some high school education or completion of high school, 31% attended some college; 26% were college graduates; 7% had some graduate training, and 19% held advanced degrees). We focus on two extreme sub-samples, daily cannabis users (who smoked 7 days per week) and lifetime abstainers. Cannabis users tended to be male, while gender was equally distributed among abstainers. Cannabis users were significantly older, and earned more money in their work. Other indices of achievement (e.g. grades) were not assessed. It is notable that the preponderance of students with no income in this sample renders findings regarding income relatively difficult to interpret accurately. See Table 1 for demographics of the two groups.

Motivation and Subjective Well-being. We employed Pearson's correlations to examine relationship among motivation and subjective well-being. Mean motivation and subjective well-being scores correlated significantly in the entire sample (r = .40, p < .001), among chronic cannabis users (r = .35, p < .001), and cannabis abstainers (r = .462, p < .01). Age did not correlate significantly with subjective well-being scores (r = .008, p > .05) or motivation scores (r = .08, p > .05).

### Motivation in users vs. non-users

In general, subjects appeared relatively motivated (*M *= 38; *SD *= 5.6). A comparison of independent means among subjects using cannabis seven days a week (*M *= 38.1; *SD *= 5.1) and non-users (*M *= 37.7; *SD *= 6.1) revealed little difference (*t*(453) = .73, *p *= .47). Missing data accounted for variation in degrees of freedom throughout analyses. We avoided using covariates or matching in initial analyses to provide maximum power for detecting group differences. A plot of the data revealed several outliers in each group. Square root transformations improved the skew, but revealed no significant differences (*t*(453) = .50, *p *= .62).

**Figure 1:**
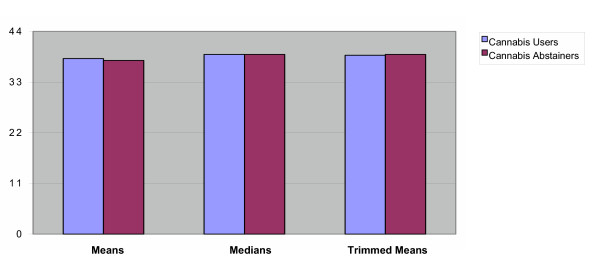
Motivation Scores Means, Medians and Trimmed Means Comparisons.

The null results from standard statistical procedures led us to turn to alternative techniques with better power. Welch's heteroscedastic means comparison revealed little difference among motivation scores for frequent cannabis users and non-users (*W*(412) = .72, *p *= .47). Plots of motivation scores revealed outliers even after standard data transformations (e.g. square roots). A comparison of median motivation among the cannabis users (*Md *= 39) and non-users (*Md *= 39) again revealed no significant difference (*p *= 1 confidence interval: -0.85, 0.85). Using Yuen's [32] method, a comparison of 20% trimmed means among cannabis users (*M*_*t *_= 38.8) and non-users (*M*_*t *_= 39.0) revealed no significant differences (*Y*(272) = .31, *p *= .76). Thus, even robust statistics with improved power failed to find differences in motivation between daily users and non-users. Measures of effect size for motivation using Cohen's d revealed a weak association (d = .06). A *Q *value of .5 suggests no real effect. As with Cohen's d, the robust analyses similarly found no significant separation of the distributions – a very weak effect [*Q *= .54; [30]]. See Figure 1.

### Subjective well-being in users Vs. non-users

The most reliable items measuring subjective wellbeing combine to form a composite 28-point scale. In general, subjects reported high levels of subjective wellbeing (*M *= 20.9; *SD *= 4.9). A comparison of independent means between daily users (*M *= 20.9; *SD *= 4.9) and non-users (*M *= 21.3; *SD *= 4.9) revealed no significant difference (*t*(426) = 1.3, *p *= .94). Again, we avoided using covariates or matching procedures to ensure maximum power in the detection of group differences. Measures of effect size suggested a weak association (d = .1). A plot of the data revealed several outliers in each group. Square root transformations improved skew [33]. A comparison of the mean square roots among the cannabis users and non-users again demonstrated no differences (*t*(426) = 1.2, *p *=.23).

A Welch's [31] comparison of heteroscedastic means revealed no significant differences (*W*(405) = 1.3, *p *=.20). Yuen's [32] comparison of 20% trimmed means among frequent cannabis users and non-users revealed no significant differences in subjective well-being scores, but did approach significance (*Y*(232) = 1.9, *p *= .05). Comparisons of medians between daily users (*Md *= 21) and non-users (*Md *= 23), however, did reveal a small but statistically significant difference (p < .001; .95 confidence interval: 1.46, 2.54). Thus, trimming 20% of the values failed to reveal inter-group differences, but effectively removing 50% of the data from each end of the distribution resulted in a significant finding.

**Figure 2:**
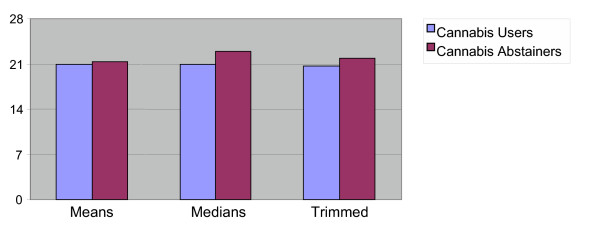
Subjective Well-being Scores Means, Medians and Trimmed Means Comparisons.

Statistical power, the likelihood of finding a meaningful effect between two distributions, increases as sample size increase. Given a large enough sample size, many small variations among groups may achieve statistical significance. Given this study's large sample size (N = 487), we employed further analyses to understand this statistically significant finding in an appropriate context. Cohen's d suggested a weak association between the variables. Similarly, a robust measure of effect size found little effect [*Q *= .55; [30]]. Thus while median differences among distributions are statistically significant, the two point difference may have little clinical significance. See Figure 2.

### Medical conditions as a source of decreased subjective wellbeing

Approximately one-third of frequent cannabis users were medical users, who often report high levels of pain, nausea and physical problems. To eliminate any possible confounds that these known medical problems could pose on measurements of subjective wellbeing, we compared non-medical cannabis users (N = 168, *M *= 21.2) with cannabis abstainers (N = 244; *M *= 21.2). A standard comparison of independent means revealed no differences (*t*(353) = .18, *p *= .86). Similarly, comparisons of heteroscedastic means revealed no significant differences in subjective well-being scores (*W*(353) = .18, *p *= .86). Frequent users (*M*_*t *_= 21.7) and abstainers (*M*_*t *_= 22.3) also demonstrated no significant differences with 20% trimmed means (*Y*(213) = 1.1, *p *= .29). A comparison of medians among frequent cannabis users (*Md *= 22) and abstainers (*Md *= 23) revealed a 1-point difference (*p *< .05, .95 confidence interval: 0.15, 1.85) on the 28-point scale. Again, Cohen's d suggested little association between the variables. A robust measure of effect size found little effect [*Q *= .51; [30]].

## Discussion

In this study, participants who used cannabis seven days a week demonstrated no difference from non-cannabis users on indices of motivation. These findings refute hypothesized associations between heavy cannabis use and low motivation. Means and median comparisons, data transformations, and robust comparisons of central locations each revealed no statistically significant differences among motivation scores despite improvements in power. Daily users reported slightly lower median subjective well-being scores (2 points less on a 28-point scale). An examination of non-medical cannabis users' and abstainers' subjective wellbeing scores revealed no mean differences using standard or robust methods, but did reveal a difference in median scores (1 point on a 28-point scale). While cannabis users were older than abstainers, age demonstrated no correlation with subjective wellbeing and a very weak correlation with motivation. The difference in age between groups actually should increase the likelihood of finding differences. For this sample size, an effect size of d = .25 should be detectable with a power of .80 and two-tailed alpha of .05. Effect size estimates using Cohen's d ranged from .06 to .1. Robust measures of effect size similarly reported no meaningful separation of distributions.

These findings suggest that chronic cannabis users need not develop motivation problems, but they report statistically significant, though small, decreases in subjective wellbeing. Post-hoc tests find that some portion of the differences in subjective wellbeing arose from medical users, whose illnesses may contribute to low subjective wellbeing more than their cannabis use. Whereas statistical power grows as sample size increases, the study's large sample size may lend statistical significance to findings that have little practical meaning to the clinician (e.g. 1 or 2 points on a 28-point scale). Nevertheless, these results do confirm the superior power of robust statistics and should encourage investigators to turn to them as superior to standard comparisons based on means. It is notable that we did not employ covariates at any point throughout the analyses (e.g. age, medical use). Researchers generally include covariates in an effort to ensure that a large effect does not arise from differences between groups on dimple demographic correlates of the dependent variable. Our analyses demonstrated no effect. Thus the inclusion of covariates would render group differences even smaller. Analyses of the data examining suppressor effects confirmed that the inclusion of age and gender as covariates would not reveal hidden effects

The relatively homogenous convenience sample used in this study warrants a brief caveat. Overall, the sample was largely Caucasian and educated. Further research on links between cannabis and motivation among a more ethnically diverse group from varying levels of education could reveal different findings. Among daily smokers, the sample was largely male. While some studies suggest that female chronic cannabis users closely resemble their male counterparts [see [34]], a greater number of female subjects could offer a more complete understanding of frequent cannabis users. Still, many laboratory-based studies on cannabis have similarly yielded largely male samples [see [35,36]], and have offered valuable information to the understanding of cannabis use.

Given the online format, the present study did not screen participants for cannabis use or any other type of heavy drug use. Furthermore, the present study did not exclude individuals using other substances. Although the study does focus expressly only cannabis use, previous research does suggest that cannabis users may misattribute low motivation to cannabis over other substances used comorbidly [e.g. alcohol; [18]]. It seems likely that individuals who have abstained from lifetime use of cannabis do not engage in use of other illicit substances. Still, the study fails to find significant differences among the two groups. Failure to control for comorbid drug use, while an important limitation, would likely contribute to intergroup differences among substance users and abstainers. Thus we believe that this limitation does not limit interpretation of the data. Even with users of other drugs included among the cannabis users, they still report motivation and well-being comparable to non-users.

Participant reactivity to questions of motivation may pose an additional confound. Despite a lack of empirical evidence supporting amotivational syndrome, the popular concept is well known among cannabis users. Perhaps cannabis users demonstrate sensitivity to questions regarding motivation, exaggerating their own motivation in an effort to defy stereotypes. In contrast, users tend to attribute low energy and motivation to cannabis even when they use alcohol problematically, so there may also be a bias for cannabis users to report lower motivation [18]. Further, collecting data via the internet may prevent some low education or low income individuals from participating. Others may feel uncomfortable reporting drug use online. Simultaneously, individuals experiencing low motivation may be more likely to participate in internet-based research rather than traveling to a laboratory.

The present findings coincide with numerous others studies that cite a lack of evidence for amotivational syndrome [e.g. [37]]. The majority of chronic cannabis users in the present sample report relatively high levels of motivation comparable to their non-using counterparts. These findings further suggest that cannabis abstainers report slightly higher median levels of subjective wellbeing. However, medical cannabis users with health problems appear to account for a substantial part of this finding. Standard and robust measures of effect size suggest little association between cannabis use and motivation or cannabis use and subjective wellbeing. Thus these findings merit further study.

A null association among frequent cannabis use and motivation contradicts prevailing popular notions. In addition to clarifying the effect of cannabis on the majority of its users, the present findings offer implications for the treatment of cannabis abuse and dependence. Debunking the myth of amotivation among all chronic cannabis users may prevent inappropriate treatments for all cannabis abusing and dependent individuals. Some problem users may be lulled into a false sense of security because they still feel motivated despite cannabis-related life problems. Researchers and clinicians may focus treatment on other constructs possibly affecting motivation among chronic cannabis users [e.g. peer group association; [37]; depression; 5]. Refuting an overarching connection between chronic cannabis use and life satisfaction promotes understanding of the severity and suffering of cannabis dependent individuals seeking treatment. Further research may also help to understand the experiences of medical cannabis users, and the effect that cannabis has in diminishing or promoting subjective wellbeing. By identifying and treating true etiological pathways underlying substance abuse and its related social problems, scientists, social policy and the public alike may adopt a more accurate and compassionate understanding of the actual lives and logic of the people in need of their help.

The absence of any link between motivation and cannabis use also has important implications for preventing substance abuse. Research informs parents and children that cannabis saps motivation [1]. It is possible that individuals' own experiences and the experiences of users they know may belie this information, leading them to question the veracity of other material presented in these programs. Thus, emphasizing a cannabis-induced amotivational syndrome in drug prevention does not have empirical support and could harm the credibility of our efforts at prevention. Honest information about the negative consequences of cannabis has the potential to improve the prevention of drug problems. Dropping references to amotivational syndrome may have considerable benefit.
